# A Pilot Study on Dietary Choices at Universities: Vending Machines, Canteens, and Lunch from Home

**DOI:** 10.3390/nu16111722

**Published:** 2024-05-31

**Authors:** Leandro Oliveira, Mona N. BinMowyna, Ibrahim Alasqah, Renata Puppin Zandonadi, Edite Teixeira-Lemos, Cláudia Chaves, Hmidan A. Alturki, Najla A. Albaridi, Fatmah Fahad Alribdi, António Raposo

**Affiliations:** 1 CBIOS (Research Center for Biosciences and Health Technologies), Universidade Lusófona de Humanidades e Tecnologias, Campo Grande 376, 1749-024 Lisboa, Portugal; 2 Polytechnic Institute of Coimbra, Coimbra Health School, Rua 5 de Outubro–S. Martinho do Bispo, Apartado 7006, 3046-854 Coimbra, Portugal; 3 College of Education, Shaqra University, Shaqra 11911, Saudi Arabia; m.mwena@su.edu.sa; 4 Department of Community, Psychiatric and Mental Health Nursing, College of Nursing, Qassim University, Buraydah 51452, Saudi Arabia; i.alasqah@qu.edu.sa; 5 School of Health, University of New England, Armidale, NSW 2351, Australia; 6 Department of Nutrition, School of Health Sciences, University of Brasilia (UnB), Campus Darcy Ribeiro, Asa Norte, Brasilia 70910-900, Brazil; renatapz@unb.br; 7 CERNAS Research Centre, Polytechnic University of Viseu, 3504-510 Viseu, Portugal; etlemos3@gmail.com; 8 ESSV, Centre for Studies in Education and Innovation (CI&DEI), Polytechnic University of Viseu, 3504-510 Viseu, Portugal; claudiachaves21@gmail.com or; 9 King Abdulaziz City for Science & Technology, Wellness and Preventive Medicine Institute—Health Sector, Riyadh 11442, Saudi Arabia; halturki@kacst.edu.sa; 10 Department of Health Science, College of Health and Rehabilitation, Princess Nourah bint Abdulrahman University, P.O. Box 84428, Riyadh 11671, Saudi Arabia; naalbaridi@pnu.edu.sa; 11 Director of Model of Care, Qassim Health Cluster, Buraydah 52367, Saudi Arabia; falribdi@moh.gov.sa

**Keywords:** vending machines, canteen, lunch, nutrition, university, students

## Abstract

Commercial environments and food acquisition methods significantly shape dietary practices and impact health. This study assesses dietary choices among Portuguese university students regarding vending machines, canteens, and lunches from home. It also evaluates their use of the university canteen and their tendency to bring lunch from home. This pilot cross-sectional study used a self-administered electronic questionnaire, made available in early 2023. Participants were recruited through snowball sampling. The study included 137 students from Portuguese higher education institutions, mainly women (74.5%), pursuing degrees or integrated Master’s degrees (83.2%), primarily in health-related fields (55.5%). The median age was 21 years (20 to 23.5 years). About 70.0% regularly consumed food from vending machines, while approximately 60.0% brought lunch from home, avoiding the canteen. Factors such as convenience (48.5%), price (47.5%), product availability (40.6%), and taste (39.6%) mainly influenced vending machine choices. Monthly, chocolates, water, coffee, cookies, treats, and soft drinks were the most commonly acquired items, with coffee being the most frequent daily purchase. These findings provide insights for creating policies and initiatives to promote healthier and more accessible food options for students and strategies to encourage positive eating behaviors.

## 1. Introduction

The university food environment is characterized by the availability of foods, preparations, and beverages (FPB); their physical and financial accessibility; the promotion of FPB; and the presence of nutritional information and advertising on campus, as well as in the surrounding areas [1]. It is known that this environment influences the eating habits of those who frequent it, potentially facilitating or hindering healthy choices [2,3]. Meals consumed at the university constitute an important part of the diet for students, teachers, and staff, highlighting the relevance of this environment in promoting healthy eating habits [1,2].

In addition to the university food environment, other factors influence the dietary choices of higher education students [2]. Studies indicate that the primary determinants of food choices among university students include lack of time, convenience, cost, taste, health status, social and physical environment, concerns about weight control [4,5,6], and even food sustainability [7,8].

One significant aspect of the university food environment is the increasing prominence of vending machines, which provide food and beverages across various settings, including higher education institutions. The presence of vending machines on university campuses offers numerous advantages for students. Firstly, they offer convenience and accessibility [9], allowing students to obtain food and drinks quickly between classes or during short breaks. Furthermore, vending machines provide diverse options, ranging from quick snacks to complete meals, catering to students’ preferences and dietary restrictions [10,11].

However, acquiring food and beverages in vending machines also has challenges. Often, the options available from these machines are commonly highly processed foods, rich in sugars, saturated fats, and sodium, which can lead to an unbalanced diet and contribute to the increase in the consumption of unhealthy foods, as evidenced in national studies conducted in Portugal [10,12,13,14] and internationally [9,11,15,16]. In addition, the lack of detailed nutritional information on product packaging and the absence of clear guidelines on healthy eating choices can make it difficult for students to make informed decisions [17].

University students are not exempt from the global health challenge of overweight and obesity, with a considerable proportion of this group being affected [18,19,20,21]. In a study encompassing 22 nations across various regions of the world with diverse economic and social conditions, statistics revealed that approximately 22% of university students are affected by overweight or obesity [22]. In Portugal, the prevalence of excess weight among higher education students reaches nearly 25% [23]. Furthermore, a report on the health and well-being behaviors of polytechnic higher education students indicates that 77.70% of students consider their diet to be reasonably healthy, while 12.90% admit to having an unhealthy diet. Conversely, 8.30% perceive their diet as very wholesome, with only 1.10% of students reporting an unhealthy diet [24]. Just as a conducive teaching environment is essential for academic success, a balanced and healthy diet is vital for students’ cognitive function, energy levels, and overall well-being. However, studies show that the regular consumption of vending machine food is associated with a greater risk of developing a variety of chronic diseases, including obesity, diabetes, cardiovascular diseases, and hypertension [25,26].

Alternatives to vending machines include canteens and bringing meals from home. Several factors influence both the choice of the canteen for meals and the overall satisfaction with the canteen’s services. Some of these factors include the quality of meals and beverages, encompassing the quality of ingredients used and the taste of the food [27,28]; the quality of service, which includes team performance, service efficiency, and the service environment [27,28]; and the waiting time for service [29], among others. Just as a conducive teaching environment is essential, having a balanced and healthy diet is equally important. Canteens should provide healthy menu options without restricting choices and should adhere to current nutritional trends [28]. However, this assertion fails to capture the complexity of the current situation, as it neglects the intricate nutritional requirements of students. These needs hint at a larger issue of nutritional imbalance, which demands deeper exploration. For example, this exploration would entail scrutinizing specific dietary deficiencies or excesses contributing to this imbalance, alongside investigating underlying factors such as socioeconomic influences, cultural norms, and the accessibility of nutritious food options [28,30,31].

Bringing lunch from home to the university is another aspect to consider in the university food environment. Healthy food consumption through packed lunches depends on various factors, including culinary skills. A systematic review of observational studies revealed that cooking at home was associated with healthier food consumption [32]. Furthermore, cross-sectional research involving university students found that more frequent cooking was correlated with an increased consumption of fruits and vegetables [33]. However, studies have indicated a lack of cooking skills and infrequent meal preparation among university students, particularly males [32,34,35]. Additionally, the decision to consume packed lunches can be influenced by various factors, such as meeting specific nutritional requirements, satisfying food preferences, overcoming time constraints for on-campus meals, identifying suitable places for university dining, and managing individual food budgets [36].

Given these challenges (e.g., the prevalence of unhealthy options in vending machines and nutritional imbalances), it is essential to implement strategies that promote healthy food choices in the university environment. For this, information about the acquisition of food and beverages in vending machines, the use of the university canteen, and the habit of taking lunch from home is necessary, especially because there is a lack of studies in Portugal in this regard. Thus, dietary choices considering vending machines, canteens, and lunch from home of a sample of Portuguese university students were investigated.

Therefore, this study is expected to provide some insights, including specific dietary interventions, aimed at enhancing the dietary habits of university students.

## 2. Materials and Methods

### 2.1. Study Design and Participants

This is a cross-sectional study in which a self-administered electronic questionnaire (Supplementary Material) was available during the first semester of 2023. The target population comprised a convenience sample of public and private Portuguese university students invited to participate in the online survey. The snowball sampling method was used to ensure a wide distribution and recruitment of participants. A web URL link was obtained for the inquiry and distributed through invitations sent by the university’s internal email system and social networking platforms. All participants completed an electronic informed consent form before participating and had the right to give up at any time during the research. The university students who consented to participate in the survey, completed the questionnaire, and submitted their answers were included in the study. All information was collected anonymously, without indicating personal data, and participants did not receive any reward. This study followed the ethical code for web-based research [37] and the recommendations of the Declaration of Helsinki [38]. The study protocol was approved by the Ethics Commission of the School of Health Sciences and Technologies of the Lusophone University (P02-23).

### 2.2. Survey Questionnaire

The questionnaire was structured into the following three main sections: sociodemographic characterization, eating habits, and acquisition of food and drinks in vending machines. This questionnaire was initially prepared and applied in Portuguese and was subsequently translated into English to facilitate its publication in international settings. This translation process involved ensuring the accuracy and clarity of the questions and responses to maintain the integrity of the data collected. The sociodemographic characterization section comprised inquiries regarding gender, age, nationality, academic level, field of study, weight, and height. The section on eating habits focused on bringing food to the university and lunch in the canteen. Thus, participants were asked if they used to take lunch from home to lunch at the university (yes or no), and if so, they were asked what the main reason was (open response). Moreover, they were asked if they used to have lunch at the university canteen (yes or no); if negative, they were asked what the main reason was (open response). Finally, participants were asked if they used to consume food or beverages purchased from vending machines. The participants who answered “no” ended the questionnaire at that point.

Regarding the section on the acquisition of food and beverages from vending machines, participants who reported purchasing food and drinks from vending machines were asked about the top three factors influencing the items they buy. These factors, adapted from a previous study [33], included: I do not know; habit or routine; the taste of food; food price; control your weight; food availability; presentation or packaging; someone else decides most of the foods that I eat; vegetarian food or other special habits; content in additives, dyes, and preservatives; my cultural, religious, or ethnic roots; ease or convenience of preparation; try to make a healthy diet; and quality or freshness of food.

Subsequently, participants were asked about their purchasing frequency of various foods and beverages that are accessible from vending machines. The products available in vending machines on the campus’s periphery were assessed and categorized into the following distinct food groups: 1. Coffee; 2. Other hot drinks with coffee; 3. Hot chocolate or other beverages with chocolate; 4. Bottled water; 5. Sparkling water; 6. Soft drinks (Coca-Cola, Sumol, Iced Tea); 7. Energy Drinks; 8. Sandwiches; 9. Cakes and pastry products; 10. Cookies; 11. Cereal bars; 12. Potato chips; 13. Solid yogurt; 14. Liquid yogurt; 15. Fresh fruit; 16. Chocolates, 17. Sweets (gums; candies); and 18. Other Snacks. Respondents were presented with a nine-point scale for indicating the frequency of acquiring these food items and beverages, encompassing the following options: “Never”, “Once a month”, “2 to 3 times a month”, “Once a week”, “2–3 times a week”, “4–6 times a week”, “Daily”, “2 to 3 times a day”, and “More than three times a day”. The questionnaire was inserted into the Google Forms platform and was distributed to university students.

### 2.3. Data Analysis

To authenticate participant responses and minimize the potential for contamination from non-human sources, data validation techniques were implemented. These methods involved identifying and eliminating any responses that appeared suspicious or inconsistent. This scrutiny included analyzing response patterns, such as unusually rapid completion times or repetitive answers, which could suggest non-human activity. Additionally, participant information was cross-referenced with demographic data to confirm the coherence of responses and ensure alignment with the characteristics of our target population.

Open-ended responses were analyzed using content analysis to extract valuable insights beyond quantitative data. This involved systematically coding and categorizing the responses to identify recurring themes and patterns. Each response was carefully reviewed to ensure comprehensive data coverage, allowing us to gain deeper insights into the participants’ choices. It is important to note that in some responses, more than one reason was identified. Additionally, the 94 different reasons were derived from the question of why they take food from home to university, while the 76 reasons referred to the question about reasons for not usually having lunch in the canteen. This process involved thoroughly reviewing all responses to identify common themes and group them according to their relevance for the analysis. Excel 365 software (for windows) was used for data management and content analysis. Two independent coders were involved in the analysis and they coded the data separately to enhance reliability. Any discrepancies or disagreements between the coders were resolved through discussion and consensus. In cases where consensus could not be reached, a third coder was consulted to adjudicate the discrepancies.

The respondents’ weight and height information were used to calculate their BMI (Body Mass Index) using the Quetlet formula, as follows: BMI = weight (kg)/height (m)^2^. The cohort was established according to the World Health Organization’s criteria, which categorizes BMI as underweight (<18.5 kg/m^2^), normal weight (18.5–24.9 kg/m^2^), overweight (25.0–29.9 kg/m^2^), or obese (≥30.0).

The statistical analysis was conducted using the Statistical Package for the Social Sciences (SPSS), version 26.0, for Windows. Categorical variables were summarized in counts and percentages. The Kolmogorov–Smirnov test was employed to assess variable distributions. The chi-square test or Fisher’s exact test was used to determine the variables’ independence, while the Mann–Whitney test was used to compare medians in independent samples. Regarding the inquiry about the frequency of acquiring food and beverages from vending machines, specific response categories were grouped due to the dispersion of responses and the low frequency of specific scale options. This consolidation was performed as follows: “Once a month” and “2 to 3 times a month” responses were combined into a single category named “Monthly”; “Once a week”, “2–3 times a week”, and “4–6 times a week” responses were grouped into a category labeled “Weekly”; and “2 to 3 times a day” and “more than three times a day” responses were unified into the “Daily” category. This approach of consolidating responses aimed to simplify and enhance the coherence of the analysis concerning the frequency of acquiring food and beverages from vending machines. Results were considered significant at a *p*-value < 0.05.

## 3. Results

This study included 137 individuals attending Portuguese higher education institutions. Most were women (74.5%) and participated in a degree or integrated Master’s degree (83.2%) in the health area (55.5%). The participants’ median age was 21 (20; 23.5) years. About 70% consume food from vending machines, 60% typically bring lunch from home to the university, and 40% eat lunch in the institution’s canteen (Table 1).

The relationships between the questions “Do you usually take lunch from home to university?”, “Do you usually have lunch in the university canteen?”, and “Do you usually consume food or beverages from vending machines?” were examined in relation to other sociodemographic characteristics, namely gender, age, level of education, field of study, and nutritional status (normal weight versus overweight). However, these data were not presented in the tables. From this analysis, it was observed that participants enrolled in health-related courses tended to take lunch from home to university (χ^2^ = 11.1; *p* = 0.01) and usually did not have lunch in the university canteen (χ^2^ = 9.2; *p* = 0.02), unlike their counterparts (n = 55; 72.4% and n = 52; 72.4% versus n = 27; 44.3% and n = 26; 42.6%, respectively). No relationships were found between the other aforementioned variables.

The questions “Do you usually take lunch from home to university?”, “Do you usually have lunch in the university canteen?”, and “Do you usually consume food or beverages from vending machines?” were also examined for their interrelationships. It was observed that, as expected, participants who brought lunch from home did not usually have lunch in the university canteen (χ^2^ = 22.0; *p* < 0.01), unlike their counterparts (n = 60; 73.2% versus n = 18; 32.7%, respectively). There was no relationship found between the questions “Do you usually take lunch from home to university?” or “Do you usually have lunch in the university canteen?” and “Do you usually consume food or beverages from vending machines?”.

In total, 74 participants answered the question of why they take food from home to university; content analysis allowed us to extract 94 reasons, based on the following: 56.4% economic reasons (saving money, having expensive lunch in the canteen, etc.); 18.1% health-related reasons; 12.8% personal tastes; 8.5% logistics/time reasons; and 4.3% reasons related to the lack of quality of canteen meals. A total of 66 participants provided reasons for not usually having lunch in the canteen. The content analysis allowed us to extract 76 reasons, based on the following: 43.4% economic reasons (price, being expensive to have lunch in the canteen, etc.); 27.6% logistics/time reasons (including taking the home meal); 13.2% personal tastes; 10.5% health-related reasons; and 5.3% reasons related to the lack of quality of canteen meals—Table 2.

The factors that have the most significant influence on dietary choices made by university students when buying from vending machines are presented in Table 3. In general, the main factors that affect the food choice and drinks in vending machines are convenience (48.5%), price (47.5%), and product availability (40.6%), as well as the taste of food (39.6%). Male participants, those who attend healthcare courses, and those who often have lunch in the canteen reported that convenience is more influential than their counterparts. In addition, students who have lunch in the canteen considered that the content of additives, dyes, and preservatives is more critical than those who do not have lunch in the canteen. Younger participants (median age: 21; P25: 19; P75: 24) indicated that price influences food choices at vending machines more than older participants (median age: 22; P25: 20; P75 24)—*p* = 0.045. No relationship was found between the factors influencing the food choice on vending machines and whether participants brought lunch food at the university, or with nutritional status (average weight versus overweight), or with level of education (Bachelor’s or Licentiate/Integrated Master’s Degree versus other courses).

Regarding acquiring food and beverages in vending machines (Table 4), more than 90% never acquired sparkling water, energy drinks, solid yogurts, liquid yogurts, and fresh fruit. At the monthly level, the most frequently acquired foods were chocolates (47.5%), water (44.6%), coffee (34.7%), cookies (35.6%), treats (30.7%), and soft drinks (24.8). At the weekly level, the most frequently acquired foods were coffee (25.7%), bottled water (19.8%), other hot beverages with coffee (9.9%), and chocolates (9.9%). At the daily level, the most acquired food was coffee (15.8%). Next, an analysis was conducted to examine the relationship between the acquisition of food and beverages from vending machines and variables such as gender, study area, canteen lunching habits, and the practice of bringing lunch from home. Male participants were observed to have a higher frequency of acquiring coffee, water, energy drinks, potato chips, and solid yogurt from vending machines than their women counterparts. Moreover, students who regularly had lunch in the canteen displayed a greater frequency of acquiring sandwiches and hot chocolate or other chocolate beverages from vending machines compared to students who did not use the cafeteria for their meals. Health students had a lower frequency of cereal bar acquisition in vending machines than students from other areas. On the other hand, they had a higher frequency of water acquisition in vending machines than students from different areas. No differences were found in the acquisition of food and beverages in vending machines among students who had the habit of bringing lunch from home to university and those who did not have this habit.

## 4. Discussion

The present study aimed to assess the purchasing behaviors of food and beverages from vending machines among higher education students in Portugal, alongside examining their utilization of university canteens and their propensity to bring lunch from home. The study revealed that a significant proportion of participants (73.7%) bring their own lunch to the university, while over half do not utilize the university canteen. These findings are consistent with a prior study conducted in 2018 among 620 Spanish and Portuguese university students, which indicated that 83.9% of respondents used vending machines [39].

Since the economic crisis of 2013, there has been an increasing trend toward consumer rationalization and a heightened focus on managing household budgets. Consequently, approximately 31% of Portuguese consumers have reduced their frequency of dining out at restaurants. This shift has led to a rise in home-cooked meals and the prevalence of packed lunches. A study conducted in 2020 during the COVID-19 pandemic found that nearly half of the participants opted for packed lunches for meals consumed outside the home [40]. Regarding the utilization of university canteens, findings from a study conducted in Indonesia indicate that over half of university students who dine away from home prefer to have their meals in the canteen [30]. Conversely, another study conducted at Polish universities found that only 35.5% of students and staff members regularly use the cafeterias daily or at least once a week [28]. Unlike some other countries, Portugal observes a customary lunch hour [28]. However, this allocated time may not be adequate for lunch in the canteen due to logistical and time constraints. For instance, the closure of university canteens and their distance from academic buildings contribute to time spent commuting and waiting in line for meals [41]. Furthermore, the cost of a full meal in private canteens significantly exceeds that of public institution canteens (EUR 2.80) [41], potentially influencing the decision to bring lunch from home. It is noteworthy that students identified logistical, time-related, and economic factors as reasons for abstaining from using the canteen and opting to bring lunch from home. Food and beverage vending machines are prevalent, particularly on a weekly basis, akin to findings in the Caruso, Klein, and Kaye [42] study. Convenience, price, product availability, and taste emerged as primary factors influencing these choices, consistent with previous research [9,27,42,43]. Moreover, the analysis of specific demographic groups revealed notable nuances. Male participants and those enrolled in health courses have shown a greater tendency to consider convenience as an influential factor than their colleagues. This may suggest that these groups may be more oriented towards practicality and efficiency in choosing foods on sales machines. However, it is essential to mention that students from health courses should be aware of opting for healthier choices than other university students due to their knowledge about food and health.

The finding suggests that students who utilize the canteen exhibit a heightened awareness of additives, dyes, and preservatives, indicating a greater consciousness regarding the nutritional quality of food. This implies that the canteen could potentially play a significant role in promoting healthier food choices among this group of students [31]. However, no correlation was identified between the factors influencing food choices from vending machines and the practice of bringing lunch from home. This indicates that the motivations driving these two behaviors may differ and that additional factors may be influential in determining such behaviors. Finally, the absence of correlation between food choice factors and participants’ nutritional status (normal weight versus overweight) emphasizes the complexity of food decisions, the influence of multiple contextual factors, and food’s simple energy value.

It was observed that a significant portion, more than 90%, never acquired items such as sparkling water, energy drinks, solid yogurts, liquid yogurts, and fresh fruits from the vending machines. However, when considering the monthly frequency, foods such as chocolates, bottled water, coffee, cookies, sweets, and soft drinks were purchased more regularly. In the weekly context, the most common acquisitions included coffee, bottled water, and other hot beverages, along with chocolates. Daily analysis revealed that coffee stood out as the most frequently acquired item. These results are consistent with other studies reporting that the most consumed foods in the university’s food environment (including vending machines) are hot drinks (coffee, hot chocolate, etc.), bottled water, and sandwiches or hamburgers, while the least consumed are healthier options like fatty fruits and fresh fruits [3,44]. This suggests a preference for convenience and quick energy boosts over healthier choices among university students.

In a study that evaluated the quality of snacks and beverages sold in vending machines, most food items sold had a high energy density and none satisfied low sugar or high fiber content criteria [45,46]. The researchers pointed out that the shortage of time, high food prices, limited availability of healthy options, and lack of motivation were the most frequently mentioned barriers by students, as obstacles to healthy eating [46,47].

The study of the relationships between the acquisition of food and beverages from vending machines and variables such as gender, area of study, the habit of having lunch in the canteen, and bringing lunch from home has brought to light some differences. The results indicated that male students had a higher frequency of acquiring certain items, such as coffee, water, energy drinks, potato chips, and solid yogurts, than female students. According to Hasan et al. [9], male students consume more soda and energy drinks than female students. Yet, another study reveals that male students consumed significantly more energy from sugary drinks than female students [48]. The findings underscore the need to consider these differences when designing strategies to promote healthier eating habits on campus. By understanding different student groups’ specific preferences and behaviors, universities can better tailor their interventions to encourage more balanced and nutritious dietary choices. Furthermore, the frequency of purchasing products such as sandwiches and hot chocolate or other chocolate beverages from the vending machines was higher among students who had lunch in the canteen than those who did not. According to the work of Czarniecka-Skubina et al. [28], students who used the canteen often choose to buy soups (39.2%) and sandwiches (28%), i.e., meals that can be consumed relatively rapidly. Thus, the hypothesis can be that students resort to vending machines to complement the meals consumed in the canteen. In addition, health students have demonstrated specific consumer standards, with a lower frequency of cereal bar acquisition and a higher frequency of water acquisition in machines than students from other areas. These findings can be associated with variations in the demographic and economic profile [49], in addition to personal factors, such as cooking skills, level of information and perspectives, as well as social influences, such as the role of friends and norms in the purchase decision, among others [50]. These insights underscore the importance of considering diverse student needs and behaviors when designing interventions to promote healthier eating habits within the university environment.

These results provide a comprehensive overview of vending machines’ food and beverage consumption habits, highlighting purchase preferences at different time intervals and the influence of demographic and behavioral variables. Additionally, they can help understand participants’ eating standards and inform health promotion strategies and healthier eating habits.

### Strengths, Limitations, and Implications

This pilot study has several strengths. Its findings offer insights into acquiring food and beverages from vending machines, presenting intervention opportunities for institutions offering products in these machines. Additionally, the study provides the questionnaire used, enabling other researchers to replicate it and establish points of comparison with future studies. Considering changes to the food options offered in vending machines is crucial to improving consumer choices and promote healthier eating habits. Providing nutritious food options in vending machines is especially relevant, given that many participants indicated that they consume less healthy foods on a regular basis. Moreover, investing in food education to guide consumers in making healthier decisions, along with enhancing university meal spaces such as canteens to offer nutritious meals, as well as lunch spaces for students who bring lunch from home, would be important steps.

While our study provides insights, it is important to recognize several limitations. Given the utilization of an online questionnaire, several measures were implemented to ensure the authenticity of participant responses. Data validation techniques were employed to identify and remove any suspicious or inconsistent responses, including scrutinizing response patterns for signs of non-human activity. Additionally, participant information was cross-referenced with demographic data to verify response consistency and alignment with our target population’s characteristics. These measures aimed to uphold the integrity and reliability of our data, minimizing the risk of contamination from non-human responses. However, it is acknowledged that no method is entirely foolproof, and there may still be potential limitations associated with web-based survey platforms. Future research could explore additional strategies or technologies to further enhance data validity and mitigate the influence of non-human responses.

Another limitation of this study is that using a self-report questionnaire may introduce the possibility of inaccurate information due to respondent bias or error. Additionally, relying on a convenience sample limits the generalizability of our findings to other contexts within Portugal.

One additional limitation stems from our decision to restrict open-ended questions solely to students who indicated “no” to using canteens and vending machines. While this approach aimed to focus on understanding the specific factors influencing students’ decisions not to use these facilities, it may have overlooked valuable insights from students who responded affirmatively. Including perspectives from both groups could have provided a more comprehensive understanding of the factors influencing food choices among university students.

Another constraint is the absence of a comprehensive analysis of potential confounding factors, such as students’ socioeconomic status. It is known that socioeconomic status can play a crucial role in dietary choices, influencing both access to certain foods and dietary preferences. The lack of consideration of these factors may have impacted the conclusions’ accuracy.

Regarding the statistical analyses, categorical variables were summarized in counts and percentages, and the Kolmogorov–Smirnov test was used to assess variable distributions. The chi-square test or Fisher’s exact test was utilized to determine the independence of variables, while the Mann–Whitney test was employed to compare medians in independent samples. While these methods are appropriate for basic statistical analysis, they come with limitations. For instance, the Kolmogorov–Smirnov test is sensitive to sample size and may not perform well with small samples. Similarly, the chi-square and Fisher’s exact tests, while useful for categorical data, do not account for potential confounding variables. The Mann–Whitney test is non-parametric and does not provide insights into the effects of multiple variables simultaneously. Additionally, the consolidation of response categories into “Monthly”, “Weekly”, and “Daily” was necessary due to the dispersion and low frequency of specific options. This simplification aimed to enhance the coherence of our analysis, but may have resulted in a loss of detailed information regarding the frequency of acquiring food and beverages from vending machines.

To address these limitations and improve the robustness of our analysis in future studies, more sophisticated statistical methods will be incorporated. Mixed-effects models will allow data with multiple levels of variability, such as individual and group differences, to be handled, providing a more nuanced understanding of the factors influencing vending machine usage. Structural equation modeling can help explore complex relationships between variables, including mediating and moderating effects, offering deeper insights into the determinants of food and beverage choices. By employing these advanced techniques, the aim is to overcome the constraints of the current methodology and enable the accounting for of confounding factors and interactions between variables, ultimately leading to more accurate and reliable results.

Recognizing and addressing these factors in future studies is essential to ensure the validity and generalizability of the results. The inclusion of measures to control for participants’ socioeconomic status, such as family income questionnaires or parental education levels, could provide a more comprehensive and accurate understanding of the associations between dietary habits and other relevant factors. Thus, it is important to emphasize that the inadequate consideration of these factors may have limited the comprehensive interpretation of the results of this study. In future research, expanding our data collection to include qualitative insights from all respondents, irrespective of their utilization of canteens and vending machines, can significantly enhance our comprehension of students’ food choices and underlying motivations. This inclusive approach would enable a more nuanced exploration of the multifaceted factors influencing food-related decisions within university settings.

Furthermore, by delving into potential interventions, such as incorporating healthier alternatives in vending machines or implementing nutritional education programs, we can make substantial strides in promoting healthier eating habits among students. These interventions hold promise in fostering a supportive environment for making nutritious and balanced food choices, even amidst the demanding schedules of university life.

By embracing these measures, higher education institutions can play a pivotal role in advocating for and facilitating healthy eating practices among students. Ensuring access to wholesome food options and imparting nutritional knowledge not only bolsters student well-being and academic performance but also instills lifelong healthy eating habits. This proactive approach underscores the commitment of universities to the holistic development and flourishing of their student communities.

## 5. Conclusions

This pilot study offers valuable insights into the eating habits of a subset of university students in Portugal. A significant portion of higher education students in Portugal bring lunch from home to the university rather than using the canteen. Food and beverage vending machines are extensively used by university students, mainly driven by convenience, price, product availability, and flavor preferences. Economic reasons and health concerns are key factors that influence the dietary choices of this sample of students, namely concerning lunch in the canteen and taking home lunch. The most often purchased foods were chocolates, bottled water, coffee, cookies, sweets, and sodas. In addition, the frequency of vending machines varies according to gender, area of study, and canteen use. Male students tend to purchase certain items more frequently than female students, and health students exhibit distinct consumer preferences, emphasizing the influence of demographic and behavioral factors on food choices. These results could potentially provide insights for future large-scale studies aimed at informing policies and programs promoting healthy and accessible food choices for students. Additionally, gaining insight into consumer preferences could pave the way for developing effective strategies to foster healthy eating behaviors among Portuguese university students. Forthcoming investigations could delve into specific areas such as the impact of vending machine placement on food choices or the effectiveness of educational interventions in promoting healthier eating habits.

## Figures and Tables

**Table 1:** Demographic and behavioral characteristics of the university students (n = 137).

Studied Parameters	Median (P25; P75)
Age	21 (20.0; 23.5)
	n (%)
Gender	
Men	35 (25.5)
Woman	102 (74.5)
Education	
Bachelor’s or Licentiate/Integrated Master’s Degree	114 (83.2)
Master’s degree	18 (13.1)
Doctorate	2 (1.5)
Postgraduate Course *	3 (2.2)
Area of the Course	
Health	76 (55.5)
Other areas	61 (44.5)
Body mass index classification	
Underweight	6 (4.4)
Normal weight	99 (72.3)
Overweight	32 (23.4)
Do you usually take lunch from home to university?	
Yes	82 (59.9)
No	55 (40.1)
Do you usually have lunch in the university canteen?	
Yes	59 (43.1)
No	78 (56.9)
Do you usually consume food or beverages from vending machines?	
Yes	101 (73.7)
No	36 (26.3)

* Courses undertaken after completing a Bachelor’s degree, without the conferral of an academic degree. For example, this includes courses with a duration of one academic year or less. P25: 25th percentile; P75: 75th percentile.

**Table 2:** Emergent factors from responses to open-ended questions.

Emergent Factors	Definition/Description	Number of Mentions	%
Do you usually bring lunch from home to eat at the university? If YES, what is the main reason? (n = 74)			
Logistics/time reasons	I do not have a cafeteria and I save more time; because it is more practical; lack of time.	8	8.5
Economic Reasons	Having expensive lunch in the canteen; more economical; lower price; savings; be cheaper; not spending money; spend less money.	53	56.4
Health-related reasons	Lack of healthy options; specific diet; diet; I can bring healthier options	17	18.1
Personal taste	I like home food more; I prefer my food to the canteen; I usually do not like eating outside the home.	12	12.8
Quality	Quality; food quality; variety.	4	4.3
Do you usually have lunch at the university canteen? If NO, what is the main reason? (n = 66)			
Logistics/time reasons	Have lunch at home; I take food when necessary; schedule allows for lunch at home; be practical/convenient.	21	27.6
Economic Reasons	Price; being expensive to have lunch in the canteen; too expensive; to save.	33	43.4
Health-related reasons	Lack of healthy options; go on a diet.	8	10.5
Personal taste	Prefiro a minha comida (I prefer my food); food not well prepared; porque normalmente não gosto da comida (because I usually don’t like the food)	10	13.2
Quality	Quality; food quality.	4	5.3

**Table 3:** Most significant factors influencing food choices made by university students when purchasing food and beverages from vending machines (n = 101).

	Total		Gender			Course			Do You Usually Have Lunch in the University Canteen?		
	No	Yes	Men	Woman	*p*	Health	Others	*p*	Yes	No	*p*
I do not know	98 (97.0)	3 (3.0)	0 (0.0)	3 (3.8)	1.000 ^a^	1 (1.9)	2 (4.2)	0.603 ^a^	2 (4.3)	1 (1.9)	0.596 ^a^
Habit or routine	66 (65.3)	35 (34.7)	10 (43.5)	25 (32.1)	0.312 ^b^	20 (37.7)	15 (31.3)	0.494 ^b^	13 (27.7)	22 (40.7)	0.168 ^b^
The taste of food	61 (60.4)	40 (39.6)	11 (47.8)	29 (37.2)	0.359 ^b^	19 (35.8)	21 (43.8)	0.417 ^b^	17 (36.2)	23 (42.6)	0.510 ^b^
Food price	53 (52.5)	48 (47.5)	8 (34.8)	40 (51.3)	0.164 ^b^	26 (49.1)	22 (45.8)	0.746 ^b^	23 (48.9)	25 (46.3)	0.791 ^b^
Control your weight	92 (91.1)	9 (8.9)	1 (4.3)	8 (10.3)	0.680 ^a^	4 (7.5)	5 (10.4)	0.733 ^a^	4 (8.5)	5 (9.3)	1.000 ^a^
Food availability	60 (59.4)	41 (40.6)	10 (43.5)	31 (39.7)	0.749 ^b^	17 (32.1)	24 (50.0)	0.067 ^b^	19 (40.4)	22 (40.7)	0.974 ^b^
Presentation or packaging	97 (96.0)	4 (4.0)	1 (4.3)	3 (3.8)	1.000 ^a^	2 (3.8)	2 (4.2)	1.000 ^a^	2 (4.3)	2 (3.7)	1.000 ^a^
Someone else decides most of the foods that I eat	101 (100.0)	0 (0.0)	0 (0.0)	0 (0.0)	…	0 (0.0)	(0.0)	…	0 (0.0)	0 (0.0)	…
Vegetarian food or other particular habits	94 (93.1)	7 (6.9)	0 (0.0)	7 (9.0)	0.346 ^a^	3 (5.7)	4 (8.3)	0.706 ^a^	5 (10.6)	2 (3.7)	0.246 ^a^
Content in additives, dyes, and preservatives	97 (96.0)	4 (4.0)	1 (4.3)	3 (3.8)	1.000 ^a^	1 (1.9)	3 (6.3)	0.344 ^a^	4 (8.5)	0 (0.0)	0.044 ^a^*
My cultural, religious, and ethnic roots	101 (100.0)	0 (0.0)	0 (0.0)	0 (0.0)	…	0 (0.0)	0 (0.0)	…	0 (0.0)	0 (0.0)	…
Ease of convenience of preparation	52 (51.5)	49 (48.5)	16 (69.6)	33 (42.3)	0.022 ^b^*	31 (58.5)	18 (37.5)	0.035 ^b^*	17 (36.2)	32 (59.3)	0.021 ^b^*
Try to make a healthy diet	81 (80.2)	20 (19.8)	4 (17.4)	16 (20.5)	1.000 ^a^	11 (20.8)	9 (18.8)	0.801 ^b^	7 (14.9)	13 (24.1)	0.248 ^b^
Quality or freshness of food	90 (89.1)	11 (10.9)	2 (8.7)	9 (11.5)	1.000 ^a^	4 (7.5)	7 (14.6)	0.257 ^b^	8 (17.0)	3 (5.6)	0.065 ^b^

* *p* < 0.05; Gender, course, and “Do you usually have lunch in the university canteen?”: ^a^ Fisher’s or ^b^ chi-square test based on the expected frequencies in each cell. Note: The data for gender, course, and food/beverage acquisition in vending machines are only presented for participants who answered “Yes” in the total column.

**Table 4:** Acquisition of food and beverages in vending machines (n = 101).

		n	Never	Monthly	Weekly	Daily	*p*
1. Coffee	Men	23	2 (8.7)	8 (34.8)	7 (30.4)	6 (26.1)	0.031
	Woman	78	22 (28.2)	27 (34.6)	19 (24.4)	10 (12.8)	
	Total	101	24 (23.8)	35 (34.7)	26 (25.7)	16 (15.8)	…
2. Other hot drinks with coffee	Total	101	57 (56.4)	31 (30.7)	10 (9.9)	3 (3.0)	…
3. Hot chocolate or other drinks with chocolate	Lunch in the canteen	47	25 (53.2)	14 (29.8)	7 (14.9)	1 (2.1)	0.007 *
	Do not have lunch in the canteen	54	42 (77.8)	9 (16.7)	3 (5.6)	0 (0.0)	
	Total	101	67 (66.3)	23 (22.8)	10 (9.9)	1 (1.0)	…
4. Bottled water	Health	76	36 (47.4)	26 (34.2)	13 (17.1)	1 (1.3)	0.048 *
	Other	61	21 (34.4)	26 (42.6)	8 (13.1)	6 (9.8)	
	Total	101	30 (29.7)	45 (44.6)	20 (19.8)	6 (5.9)	…
5. Sparkling water	Men	23	19 (82.6)	3 (13.0)	1 (4.3)	0 (0.0)	0.024 *
	Woman	78	75 (96.2)	3 (3.8)	0 (0.0)	0 (0.0)	
	Total	101	94 (93.1)	6 (5.9)	1 (1.0)	0 (0.0)	…
6. Soft drinks (Coca-Cola, Sumol, Iced Tea)	Total	101	74 (73.3)	25 (24.8)	2 (2.0)	0 (0.0)	…
7. Energy Drinks	Men	23	18 (78.3)	1 (4.3)	4 (17.4)	0 (0.0)	<0.001 *
	Woman	78	78 (100.0)	0 (0.0)	0 (0.0)	0 (0.0)	
	Total	101	96 (95.0)	1 (1.0)	4 (4.0)	0 (0.0)	…
8. Sandwiches	Lunch in the canteen	47	32 (68.1)	11 (23.4)	4 (8.5)	0 (0.0)	0.036 *
	Do not have lunch in the canteen	54	46 (85.2)	7 (13.0)	1 (1.9)	0 (0.0)	
	Total	101	78 (77.2)	18 (17.8)	5 (5.0)	0 (0.0)	…
9. Cakes and pastry products	Total	101	75 (74.3)	21 (20.8)	5 (5.0)	0 (0.0)	…
10. Cookies	Total	101	61 (60.4)	36 (35.6)	4 (4.0)	0 (0.0)	…
11. Cereal bars	Health	76	69 (90.8)	7 (9.2)	0 (0.0)	0 (0.0)	0.040 *
	Another course	61	48 (78.7)	10 (16.4)	3 (4.9)	0 (0.0)	
	Total	101	83 (82.2)	15 (14.9)	3 (3.0)	0 (0.0)	…
12. Potato chips	Men	23	16 (69.6)	5 (21.7)	2 (8.7)	0 (0.0)	0.023 *
	Woman	78	69 (88.5)	9 (11.5)	0 (0.0)	0 (0.0)	
	Total	101	85 (84.2)	14 (13.9)	2 (2.0)	0 (0.0)	
13. Solid yogurt	Men	23	20 (87.0)	1 (4.3)	1 (4.3)	1 (4.3)	0.039 *
	Woman	78	76 (97.4)	2 (2.6)	0 (0.0)	0 (0.0)	
	Total	101	96 (95.0)	3 (3.0)	1 (1.0)	1 (1.0)	
14. Liquid yogurt	Total	101	94 (93.1)	5 (5.0)	2 (2.0)	0 (0.0)	…
15. Fresh fruit	Total	101	94 (93.1)	6 (5.9)	0 (0.0)	1 (1.0)	…
16. Chocolates	Total	101	42 (41.6)	48 (47.5)	10 (9.9)	1 (1.0)	…
17. Sweets (gums; candies)	Total	101	65 (64.4)	31 (30.7)	4 (4.0)	1 (1.0)	…
18. Other Snacks	Total	101	78 (77.2)	19 (18.8)	4 (4.0)	0 (0.0)	…

* *p* < 0.05; Men/Woman, Lunch in the canteen/Do not have lunch in the canteen, and Health/Other course: Mann–Whitney test. Only detailed data are presented for the results where significant differences exist.

## Data Availability

Data are contained within the article.

## Supplementary Materials

The following supporting information can be downloaded at: https://www.mdpi.com/article/10.3390/nu16111722/s1, Questionnaire applied to participants.
