# Supplementary material for: A Pilot Study on Dietary Choices at Universities: Vending Machines, Canteens, and Lunch from Home
Source: Nutrients. 2024 May 31;16(11):1722. doi: 10.3390/nu16111722 (PMC11174703; doi:10.3390/nu16111722)
Supplement: Supplementary file 1 [file nutrients-16-01722-s001.zip › nutrients-3020925-supplementary.pdf]

Supplementary Material

Questionnaire applied to participants

Caracterização sociodemográfica/ Sociodemographic characterization

1. Qual é o seu gendero?/ What is your gender?
Marcar apenas uma opção/ Select only one option
( )Feminino/ Woman
( )Masculino / Men
( )Outra/ Other:____________________

2. Qual é a sua idade (anos)?/ What is your age (years)? ________

3. Qual é a sua nacionalidade? / What is your nationality?
Marcar apenas uma opção/ Select only one option
( ) Portuguesa/ Portuguese
( ) Outra/ Other: _____________

4. Qual é a sua Região de residência (NUTS II)?/ What is your region of residence (NUTS II)?
Marcar apenas uma opção/ Select only one option
( ) Norte/ North
( ) Centro/ Center
( ) Alentejo/ Alentejo
( ) Área Metropolitana de Lisboa/ Lisbon Metropolitan Area
( ) Oeste e Vale do Tejo/ West and Tagus Valley
( ) Península de Setúbal/ Setúbal Peninsula
( ) Algarve/ Algarve
( ) Região Autónoma da Madeira/ Madeira Autonomous Region
( ) Região Autónoma dos Açores/ Azores Autonomous Region

5. Qual é a sua altura (m)?/ What is your height (m)? ___________

6. Qual é o seu peso (kg)?/ What is your weight (kg)? ____________

7. Qual é o ciclo de estudos frequenta?/ What level of studies are you attending?
Marcar apenas uma opção/ Select only one option
( ) Licenciatura ou Mestrado Integrado/ Undergraduate or Integrated Master's degree
( ) Mestrado/ Master's degree
( ) Doutoramento / Doctorate
( ) Pós-graduação/ Postgraduate
( ) Outra/ Other: _______________________

8. Qual é a área do curso que frequenta? / What is the field of study you are enrolled in?
Marcar apenas uma opção/ Select only one option
( ) Saúde/ Heath
( ) Ciências Económicas e das Organizações/ Economic and Organizational Sciences
( ) Comunicação, Arquitetura, Artes e Tecnologias de Informação/ Communication, Architecture, Arts, and Information Technologies
( ) Educação Física e Desporto/ Physical Education and Sports
( ) Ciências sociais, Educação e Administração/ Social Sciences, Education, and Administration
( ) Direito/ Law
( ) Engenharia/ Engineering
( ) Medicina/ Medicine
( ) Veterinária/ Veterinary
( )Serviço Social/ Social Service
( ) Outra/ Other: ____________________________

9. A instituição de ensino superior que frequenta é/ The higher education institution you attend is:

Marcar apenas uma opção/ Select only one option

( ) Pública Estatal / Public State (e.g. UL, UNL, UP, ESEL...)
( ) Pública - Não Estatal/ Public - Non-State (e.g. UCP)
( ) Privada/ Private (e.g. ULusófona, Egas Moniz...)

Hábitos alimentares/ Eating habits

1. Com que frequência realiza as seguintes refeições?/ How often do you have the following meals? (marcar apenas uma opção por linha/ select only one option per line)

	Nunca/ Never	1 vez por semana/ 1 time per week	2 vezes por semana/ 2 times per week	3 vezes por semana/ 3 times per week	4 vezes por semana/ 4 times per week	5 vezes por semana/ 5 times per week	6 vezes por semana/ 6 times per week	Todos os dias/ Every day
Pequeno-almoço/ Breakfast								
Merenda da manhã/ Mid-morning snack								
Almoço/ Lunch								
Merenda da tarde / Afternoon snack								
Jantar / Dinner								
Ceia / Supper								
Petiscar entre as refeições/ Snacking								

betwe en meals								

2. Com que frequência costuma consumir os seguintes alimentos como **merenda/ lanche**?/ How often do you usually consume the following foods as a snack? (marcar apenas uma opção por linha/ select only one option per line)

	Nunca ou menos de 1 vez por mês/ Never or less than once a month	2 a 3 vezes por mês/ 2 to 3 times a month	1 a 2 vezes por semana/ 1 to 2 times a week	3 a 4 vezes por semana/ 3 to 4 times a week	5 a 6 vezes por semana/ 5 to 6 times a week	1 vez por dia/ Once a day	2 a 3 vezes por dia/ 2 to 3 times a day	Mais de 3 vezes por dia/ More than 3 times a day
Fruta/ Fruit								
Iogurte meio gordo/ Low-fat yogurt								
Iogurte magro/ Fat-free yogurt								
Sandes (queijo, fiambre, manteiga etc.)/ Sandwich (cheese, ham, butter, etc.)								
Chocolates/ Chocolate bars								
Bolos e produtos de pastelaria / Cakes and pastries								
Snacks salgados (batata frita, etc.) / Savory snacks (potato chips, etc.)								
Bolachas doces /								

Sweet biscuits								
Bolachas salgadas / Savory biscuits								
Bolachas dietéticas (integrais, ricas em fibras etc.) / Dietary biscuits (wholegrain, high-fiber, etc.)								
Barras de cereais / Cereal bars								
Outro/ Other								

3. Com que frequência costuma preparar as suas refeições (cozinhar)?/ How often do you usually prepare your meals (cook)?
Marcar apenas uma opção/ Select only one option

( ) Nunca/ Never
( ) 1 vez por semana/ Once a week
( ) 2 vezes por semana/ Twice a week
( ) 3 vezes por semana/ Three times a week
( ) 4 vezes por semana/ Four times a week
( ) 5 vezes por semana/ Five times a week
( ) 6 vezes por semana/ Six times a week
( ) Todos os dias/ Every day

4. Costuma levar o almoço de casa para almoçar na faculdade?/ Do you usually bring lunch from home to eat at the university?
Marcar apenas uma opção/ Select only one option
( ) Sim/ Yes
( ) Não/ No

4.1. **Se SIM**, qual a principal razão?/ If YES, what is the main reason? ________________

5. Costuma almoçar na cantina da faculdade?/ Do you usually have lunch at the university canteen?
Marcar apenas uma opção/ Select only one option
( ) Sim/ Yes
( ) Não/ No

5.1. **Se NÃO**, qual a principal razão?/ If NO, what is the main reason?_____________________

6. Costuma consumir alimentos ou bebidas adquiridos em máquinas de venda automática?/ Do you usually consume food or drinks purchased from vending machines?
Marcar apenas uma opção/ Select only one option
( ) Sim/ Yes
( ) Não/ No

Aquisição de Alimentos e Bebidas em Máquinas de Venda Automática/ Acquisition of Food and Beverages from Vending Machines

1. Existem muitas razões para escolher os alimentos que comemos. De acordo * com esta lista, na sua opinião, quais são os **3 fatores** que têm maior influência na sua escolha dos alimentos que compra nas **máquinas de venda automática**?/ There are many reasons for choosing the foods we eat. According to this list, in your opinion, what are the 3 factors that have the greatest influence on your choice of food you buy from vending machines?

Escolha apenas 3/ Choose just 3.

( ) Não sabe / Don't know
( ) Hábito ou rotina / Habit or routine
( ) O sabor dos alimentos / Taste of the food
( ) Preço dos alimentos / Price of the food
( ) Controlar o seu peso / Weight control
( ) Disponibilidade de alimentos / Food availability
( ) Apresentação ou embalagem / Presentation or packaging
( ) Outra pessoa decide a maior parte dos alimentos que eu como / Someone else decides most of the food I eat
( ) Alimentação vegetariana ou outros hábitos especiais / Vegetarian diet or other special habits
( ) Conteúdo em aditivos, corantes e conservantes / Content of additives, colorants, and preservatives
( ) As minhas raízes culturais, religiosas ou étnicas / My cultural, religious, or ethnic roots
( ) Facilidade ou conveniência de preparação / Ease or convenience of preparation
( ) Tentar fazer uma alimentação saudável / Trying to eat healthily
( ) Qualidade ou frescura dos alimentos / Quality or freshness of the food

2. Para que refeições costuma utilizar as máquinas de venda automática?/ For which meals do you usually use vending machines?

Marcar tudo o que for aplicável/ Check all that apply.

( ) Pequeno-almoço / Breakfast
( ) Merenda da manhã / Morning snack
( ) Almoço / Lunch
( ) Merenda da tarde / Afternoon snack
( ) Jantar / Dinner
( ) Ceia / Supper
( ) Petiscar entre as refeições principais / Snacking between main meals

3. Quais alimentos/ bebidas e com que frequência os/ as costuma adquirir nas máquinas de venda automática?/ What foods/drinks do you usually buy from vending machines and how often?

Marcar apenas uma opção por linha/ select only one option per line

	Nunca / Never	1 vez por semana / Once a week	2 a 3 vezes por semana / 2 to 3 times a week	4 a 6 vezes por semana / 4 to 6 times a week	Diariamente/ Daily
Café/ Coffee					
Outras bebidas quentes com café/ Other hot coffee drinks					
Chocolate quente ou outras bebidas com chocolate/ Hot					

chocolate or other chocolate drinks					
Água/ Water					
Água com gás/ Sparkling water					
Refrigerante/ Soft drink					
Bebidas energéticas/ Energy drinks					
Sandes/ Sandwiches					
Bolos e produtos de pastelaria/ Cakes and pastries					
Bolachas/ Biscuits					
Barras de cereais/ Cereal bars					
Batatas fritas/ Potato chips					
Iogurte sólido - Solid yogurt					
Iogurte líquido/ Liquid yogurt					
Fruta fresca/ Fresh fruit					
Chocolates/ Chocolates					
Guloseimas (gomas, pastilhas)/ Sweets (gummies, chewing gum)					
Outros snacks/ Other snacks					

Note: The questionnaire was distributed in Portuguese, and a free translation was made for publication purposes.
